# Lignocellulosic Biomass-Derived Nanocellulose Crystals as Fillers in Membranes for Water and Wastewater Treatment: A Review

**DOI:** 10.3390/membranes12030320

**Published:** 2022-03-11

**Authors:** Olawumi O. Sadare, Kelvin O. Yoro, Kapil Moothi, Michael O. Daramola

**Affiliations:** 1 Department of Chemical Engineering, Faculty of Engineering the Built Environment, Doornfontein Campus, University of Johannesburg, P.O. Box 17011, Johannesburg 2028, South Africa; kmoothi@uj.ac.za; 2 Energy Technologies Area, Lawrence Berkeley National Laboratory, 1 Cyclotron Road, Berkeley, CA 94720, USA; kelvin.yoroo@gmail.com; 3 Department of Chemical Engineering, Faculty of Engineering, Built Environment and Information Technology, University of Pretoria, Hatfield, Pretoria 0028, South Africa; Michael.daramola@up.ac.za

**Keywords:** cellulose nanocrystals, circular economy, life cycle assessment, membrane applications, water and wastewater treatment, waste biomass

## Abstract

The improvement of membrane applications for wastewater treatment has been a focal point of research in recent times, with a wide variety of efforts being made to enhance the performance, integrity and environmental friendliness of the existing membrane materials. Cellulose nanocrystals (CNCs) are sustainable nanomaterials derived from microorganisms and plants with promising potential in wastewater treatment. Cellulose nanomaterials offer a satisfactory alternative to other environmentally harmful nanomaterials. However, only a few review articles on this important field are available in the open literature, especially in membrane applications for wastewater treatment. This review briefly highlights the circular economy of waste lignocellulosic biomass and the isolation of CNCs from waste lignocellulosic biomass for membrane applications. The surface chemical functionalization technique for the preparation of CNC-based materials with the desired functional groups and properties is outlined. Recent uses of CNC-based materials in membrane applications for wastewater treatment are presented. In addition, the assessment of the environmental impacts of CNCs, cellulose extraction, the production techniques of cellulose products, cellulose product utilization, and their end-of-life disposal are briefly discussed. Furthermore, the challenges and prospects for the development of CNC from waste biomass for application in wastewater treatment are discussed extensively. Finally, this review unraveled some important perceptions on the prospects of CNC-based materials, especially in membrane applications for the treatment of wastewater.

## 1. Introduction

The interdisciplinary, novel and high-value development of green separation techniques is required to address the sustainability challenges in existing membrane technology [1]. Owing to its flexibility and vast potential to develop scientific and technological advances, nanotechnology is perhaps one of the most commonly used approaches that have attracted emergent scientific and public interest to offer solutions that can encompass the restrictions of membrane sustainability technology [1]. The evolutions and developments explored in this field have led to the development of wastewater treatment as an inexpensive and viable alternative [2]. In this regard, the model shift made by the current methodologies and technological innovation based on the new cutting edge in facilitating nanotechnology and nanoscience has encouraged the growth of viability by minimizing the use of energy, chemicals, and membrane materials. This results in reduced sources of environmental problems in the long run [3]. High selectivity, a small plant footprint, environmental friendliness, and low energy requirements are the fundamental features of membrane technologies. This may allow extensive applications of these technologies in the treatment of surface water without the challenges of traditional treatment processes [4]. 

Nanotechnology’s application in the development of nano-supported membranes is expected to proffer solutions to the drawbacks of the existing materials and processes, and to optimize the performance of the processes employed in wastewater treatment. Most essentially, astounding results have been recorded through these great feats, including nano-supported membranes that combine both high performance and high rejection to meet the rising industry and research prospects [5]. The exceptional indication of the flexibility of nanomaterials and their nanocomposites to offer an alternative direction toward sustainable development has attracted the interest of research communities. This provides the key incentive for the stakeholders and industries to be optimistic regarding the capability of these new-generation technologies to make a huge impact for up-to-date, inexpensive and environmentally sound solutions for the water scarcity crisis. Despite this, the revolution of the era of nanotechnology has the potential to bring the capability of membrane science and engineering a giant step towards the advancement of wastewater treatment technology [6]. This is a drive to address Sustainable Development Goal 6.3 (SDG 6.3) of the United Nations, which aims to treat wastewater and ensure the quality of water and sanitation by 2030. 

About 33% of the world’s population does not have access to clean water, and it is anticipated that by 2025, the statistics will have risen to 66% [1]. The scarcity of clean water affects every aspect of human life throughout the world, with the prevalent adverse effects being on the least technologically advanced countries and relegated populations. About 36% of the world’s population resides in water-scarce areas, with over two billion people left with no alternative than to drink unclean water. The most common threat to the destruction of the ecosystem is water pollution, resulting in the loss of biodiversity, with irrevocable consequences. Water scarcity is anticipated to dislodge over 700 million people by 2030, while the means of support of one billion people living in 100 countries around the world will be put in jeopardy by 2050. Regardless of these threats, our society has very insufficient incentives to consume less water, maintain quality water, or assign capital and funds to ecosystems or social goals [1]. 

The wastewater discharged by the paper, dye, textile, printing, cosmetic, and food industries is comprised of notable amounts of dyes, which results in several challenges after being combined with water bodies. These challenges include increased chemical oxygen demand, decreased light penetrability, and visibility. In addition, most of these dyes are toxic, carcinogenic, and mutagenic to aquatic organisms. Therefore, releasing them into the environment could pose severe dangers to aquatic life, human health, and water resources [7,8,9]. Therefore, urgent intervention is required to address this challenge. 

The improvement of membrane materials for use in the treatment of wastewater has attracted the attention of numerous researchers in recent times. However, several challenges such as poor performance, short lifespan, fouling ability, and poor water flux are limiting the utilization of membranes in wastewater treatment. Numerous efforts have been made to enhance the longevity and performance of composite membranes by incorporating different materials [10]. In recent times, nanomaterials such as graphene oxide (GO), carbon nanotubes (CNTs), titanium oxide, and silica have been utilized in the fabrication of nanocomposite membranes, resulting in enhanced fouling resistance, permeability, hydrophilicity, and membrane selectivity. Reports have shown that there is an improvement in water flux upon the addition of nanomaterials, without compromising the rejection of pollutants. However, the application of membranes containing any of these nanomaterials comes with environmental and health concerns [11,12]. Hence, cellulose nanomaterials offer a suitable alternative to these environmentally hazardous nanomaterials [10,12,13]. 

According to market analysis indices, the nano-cellulose market is forecast to be worth USD 783 Million by 2025 [14]. The increasing demand and the employment of innovative applications of nano-cellulose have propelled industries and researchers to further exploit nanocellulose development [15]. Nanomaterials obtained from cellulose—such as cellulose nanofiber (CNF), cellulose nanocrystals (CNCs), and bacterial cellulose (BC)—have attracted the attention of many researchers owing to their large specific surface area, improved mechanical strength, and enhanced crystallinity. They agree with the trend in surface modification, resulting in high performance for numerous sustainable applications in nanotechnology [16,17,18]. Amongst them, cellulose nanocrystals (CNCs) have become the most viable materials in the field of cellulose science and technology owing to their easy extraction techniques, enhanced strength, biodegradability, elastic modulus, large specific surface area, good reaction activity, improved adsorption capability, and crystallinity [19,20,21]. During CNC preparation, acid hydrolysis of the microcrystalline cellulose (MCC) is commonly done to remove the amorphous region of cellulose fibers [22]. The utilization of waste biomass as raw materials in the synthesis of cellulose nanocrystals could assist in attaining zero-waste generation goals, which are also regarded as a circular economy, alleviating waste management challenges and environmental problems. 

Recently, most research efforts in CNCs have shifted attention to their biomedical applications, such as biological imaging, antibacterial applications, tissue engineering, drug delivery, and vascular transplantation [23,24,25]. Furthermore, the applications of CNCs in energy storage, biosensors, electronics, catalysis, and membrane technology have also been extensively studied. Carpenter et al. [12] reviewed cellulose nanomaterials as an alternative viable material to CNT in wastewater treatment applications. Furthermore, Sharma et al. [26] reviewed the application of lignocellulosic biomass-derived nanocellulose for wastewater remediation, with a special focus on the remediation of heavy metals. Amusa et al. [27] also appraised the separation of CO_2_ using polymeric mixed matric membranes (MMMs) containing pretreated lignocellulosic biomasses. However, only a few comprehensive reviews in the field of nanocellulose-based membrane technology for the treatment of water and wastewater are presented in the literature [28,29,30]. 

**Figure 1:**
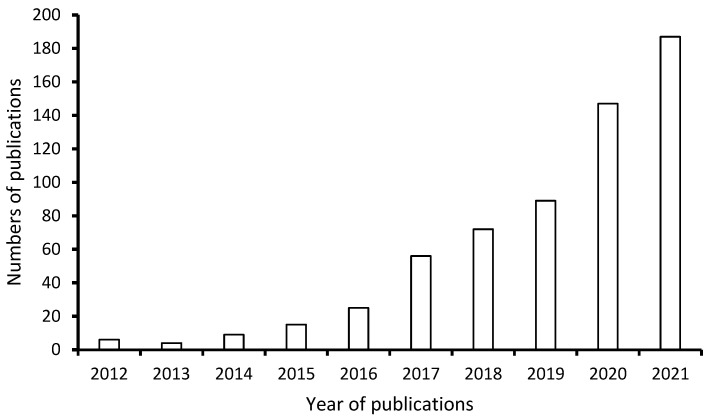
Scientific publications on nanocellulose crystal-based membranes for wastewater treatment (data extracted from www.sciencedirect.com on 14 July 2021 from Science direct, using the keywords “nanocellulose crystal”, “membrane”, and “wastewater treatment”).

Against this background, this study comprehensively reviews the membrane applications of cellulose nanocrystals derived from waste lignocellulosic biomass from the perspective of a circular economy for the treatment of wastewater to aid the trend of the lab-scale development of CNC-based membranes to an industrial scale with a good market value. The life cycle analysis of the cellulose was also highlighted to address the challenges arising from a lack of accurate evaluation due to insufficient laboratory data that encompasses all of the effects of cellulose products on different environmental aspects such as human health and air emissions. Figure 1 shows the increasing number of publications on nanocellulose applications per year, from six articles in 2012 to 187 in 2021, indicating the focus of this review on the current trends in the utilization of nanocellulose crystals in membrane applications for wastewater treatment. 

This review presents the current developments and future perceptions on the improvement of membranes using nanocellulose crystals derived from waste lignocellulosic biomass for applications in water and wastewater treatment. Recent advances and findings have not been adequately reported in the previous research. Therefore, this review succinctly provides some of the most recent applications of nanocellulose (NC), particularly cellulose nanocrystals (CNC), and their present applications in membrane technology for wastewater treatment. Firstly, summaries of cellulose, the classifications of nanocellulose, and the synthesis of cellulose nanocrystals from various waste biomasses are highlighted. The surface modification of cellulose via various routes—such as physical, chemical, and enzymatic routes—is discussed in detail, along with their characteristic properties. The circular economy of cellulose is presented, while more light is shed on recent developments and current investigations on the utilization of nanocellulose. In addition, this review considers the life cycle evaluation of cellulose products, and the proposed approaches to decreasing the energy demand and the production of a low-carbon budget for cellulose products are provided. Finally, the review concludes with remarks on the challenges and directions of future research in the field of nanocellulose-based membranes. In conclusion, speculations on future outlooks are provided. It is anticipated that this review will lead to a new direction for nanocellulose preparation and the design and development of novel nanocellulose-based materials for extensive cutting-edge applications. 

### 1.1. The Conceptualization of the Circular Economy and Waste Lignocellulosic Biomass

As a result of the abatement of natural resources and accelerating greenhouse effects, the global awareness of the utilization of biomass as an alternative material to fossil resources for a number of applications is increasing. Approximately 15 billion tons of biomass were utilized for food, animal feed, and processing, while the energy industry expended 2.1 billion tons in 2011 [31]. It is proposed that 55% of all energy expended by 2050 in the European Union will come from renewable sources [32]. As a result, this will increase the utilization of biomass by its incineration to generate bioenergy, and will thus create a considerable rise in the quantity of biomass waste generated [31]. Within the framework of the circular economy, the economic and environmental impact of such social conduct is objectionable. Therefore, the valorization of this waste, as established in the European Union Directive 2008/98/EC (2008/98/EC) on waste and the 3Rs norm (reduction, reuse, and recycling), is encouraged. 

**Figure 2:**
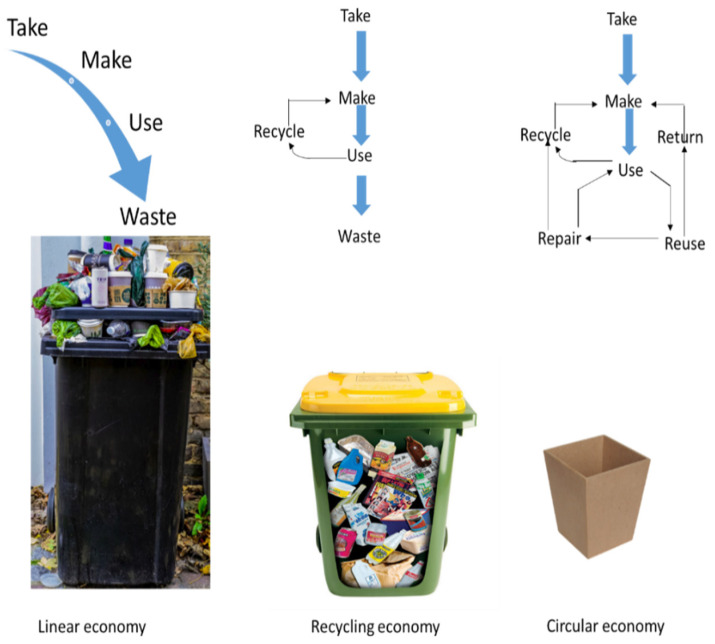
Circular economy of waste products (modified from the Asian Development Plan, 2020).

Ever-changing policies and guidelines are driving the minimization of waste generation, and are promoting the bio-based economy. The only promising solution to the valorization of waste is the integration of approaches that create products and materials in a manner that is more circular and sustainable to achieve sustainable development goals. Furthermore, comprehensive studies on the recovery of various products are required to address the present challenges of waste biomass. Furthermore, the shift from a linear economy to a more circular economy by achieving sustainable development goals is promoted as shown in Figure 2.

Biomass waste is a feedstock that can be derived from a wide range of sources. Considerably greater significance is expected to be given to reuse and recycling if waste is to become a resource to be fed back into the economy as raw material, as stated by the European Commission in 2018 [32]. The concept of a “zero waste economy” encourages the utilization of waste biomass as raw materials, in substitution of fossil resources, to be reused and modified into new molecules. There are three main types of biomass waste: agricultural residue, forest residue, and industrial residue from the wood industry, the food industry, the pulp and paper industry, and municipal solid waste and sewage. The production of agricultural waste worldwide is valued at approximately 998 million tonnes yearly [33]. Agricultural residues are categorized into four main groups, namely crop residues (bagasse, leaves, peel, straw, stem, shell, stalk, husk, pulp, and stubble, etc.), animal waste (carcasses and excreta), processing waste (packaging material), and hazardous waste (insecticides, pesticides, and herbicides) [34]. The technology of the application of agriculture waste must comply with two requirements: the prompt utilization of the waste and/or storage in a condition that prevents decay. 

Some advantages of the circular economy include attaining zero-waste generation goals, alleviating waste management challenges, reducing waste management-related costs, assisting in sustainable material and chemical productions, and encouraging the circular bio-economy. Thus, the application of sustainable technologies to recover more valued products from waste biomass assists in the reduction of environmental problems. The idea of a circular economy or zero-waste process in sustainable waste control keeps resources within the economy the moment a product has reached the end of its life [35]. These products can be efficiently used all over again; therefore, owing to the increasing utilization of cellulose products currently, one of the important domains of this review is the effort to integrate the circular economy and the appraisal of its environmental effects arising from cellulose extraction, the fabrication approaches of cellulose products, cellulose product usage, and their end disposal. 

There are major challenges to the practical application of circular economy concepts in terms of environmental sustainability. Owing to the second law of thermodynamics—entropy, as stated by Georgescu-Roegen [36]—recycling will constantly need energy, and there will always be incomplete waste and side-product generation. This could be attributed to increasing entropy and decreasing energy. This, of course, is true. Degenerated materials are lost in the environment, and their recovery is always difficult. The exploration, assembly, and recovery would require huge amounts of energy [37]. Hence, according to Georgescu-Roegen, total recycling is impossible [36]. For instance, in the use of residues cut from the forest as a source for renewable energy and an alternative to the combustion of fossil fuel, the nutrient-rich portions of the trees, bark, twists, branches, and needles are detached from the forest ecosystem, where they would assist in the ecosystem’s wellbeing, biodiversity and forest development [38]. Energy is required for this action energy. Furthermore, the machine utilized for this industrial process requires energy and materials, resulting in waste and by-product generation. Nevertheless, a case-by-case analysis is required for the sustainable contribution of circular economy projects.

### 1.2. Configuration of Nanostructured Cellulose Materials

Cellulose is the most readily available and extensively distributed natural macromolecule on earth and is affordable [39]. It is a macromolecule polysaccharide that comprises D-glucose, in which the link between the glucose molecules is through β (1→4) glycosidic bonds [40]. The presence of a large number of active hydroxyl groups in the molecular chains allows the easy formation of intramolecular and inter-molecular hydrogen bonds. The β (1→4) glycosidic bonds can be broken through acid or alkali- pre-treatment and subsequently enzymolysis for CNC formation in a strongly acidic environment [41,42]. 

**Figure 3:**
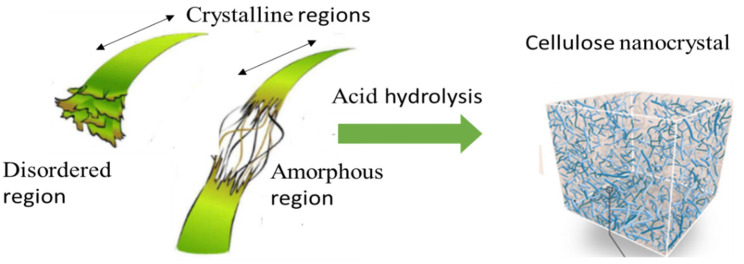
A schematic diagram of the reaction between cellulose and strong acid to obtain a nanocellulose crystal (adapted and modified from Isogai et al [43]).

Each of the glucosyl units consists of one primary hydroxy group (C_6_–OH) and two secondary hydroxy groups (C_2_–OH and C_3_–OH). The hydrophilic nature of cellulosic materials is promoted by the hydroxy groups. Furthermore, abundant C-H groups in the axial direction of the glucosyl units in cellulose play a major role in the hydrophobic interactions between cellulose molecules, and also between cellulose and other hydrophobic compounds in water [43], as depicted in Figure 3. 

**Figure 4:**
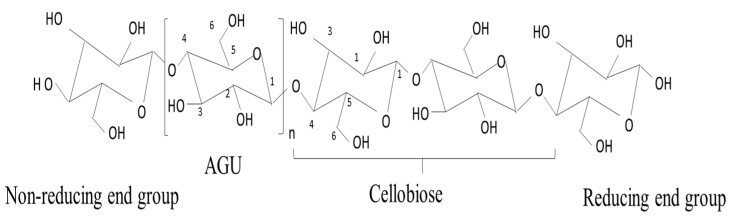
Structure of cellulose with the carbon atoms numbered, showing the repeating cellobiose unit in cellulose (adapted and modified from Marinho [53]).

**Figure 5:**
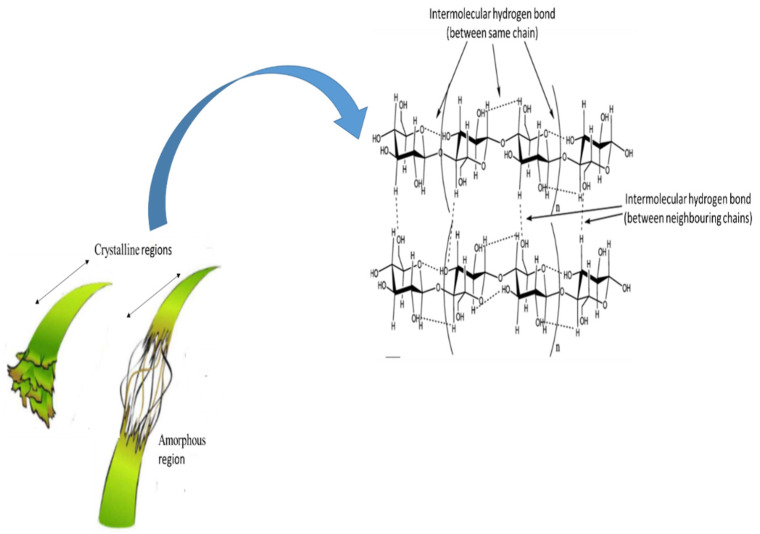
Intramolecular and intermolecular hydrogen bonds in crystalline cellulose (adapted and modified from Hasan et al. [54]).

The cellobiose units generated are bundled together to create a crystalline structure of cellulose known as basic fibrils (Figure 4). The basic fabrils are linked together to create micro-fibrils, which eventually form macro-fibrils or cellulosic fibers. The specific properties of cellulose—such as insolubility in most aqueous solvents, hydrophilicity, the ease of chemical functionalization, infusibility, and chirality are transferred by the intra- and intermolecular chemical groups [14]. The characteristics of cellulose strictly depend on the degree of polymerization and the length of the polymer chain. Natural cellulose is comprised of both disordered (amorphous) and ordered (crystalline) regions. The crystallinity usually varies from 40 to 70% based on the natural source and the methods of extraction. In comparison, the amorphous regions have a lower density than the crystalline ones, and they are susceptible to reacting with other molecular groups [44,45]. Generally, crystalline regions are more resistant to mechanical, chemical, and enzymatic treatments compared to amorphous regions. The crystalline regions of CNC are formed via the intramolecular and intermolecular hydrogen bonds of cellulose macromolecules, as shown in Figure 5. Cellulose can exist as different polymorphs, depending on its molecular orientations, van der Waals, intramolecular and intermolecular interactions, and extraction and method of treatment, which can be modified using thermal or chemical treatments [46]. CNC is extensively applied in food [47], adsorption [48,49], and medical materials [50], as well as membrane applications [51,52].

### 1.3. Isolation of Nanocellulose Crystal from Waste Lignocellulosic Biomass Sources

The demand for cellulose fibers is anticipated to exceed the existing supply in the near future, considering the ever-growing research interest that has resulted in the development of new cellulose-based products [55,56]. Owing to the ready availability and cost-effective extraction that allows large-scale production, wood pulp is the most prevalent source of cellulose. However, the methods of preparation are costly, with low-yield production, signifying a major drawback when large-scale production is proposed. Conversely, some environmental challenges, such as forest devastation and the resulting contribution to global warming are associated with the preparation processes, have motivated researchers to find eco-friendly and biocompatible materials as viable substitutes to wood-derived cellulose [57]. In a drive to search for alternative wood-derived cellulose sources, cellulose obtained from bacteria and chemical synthesis is currently receiving much attention from the advanced industries. 

Non-wood cellulosic biomass sources, such as industrial and agricultural wastes, provide a unique alternative to traditional wood pulp. In addition to being cheap, they are abundant in nature and renewable. Primarily, these non-wood sources are sustainable materials, as a result of their usual higher hemicellulose and lower lignin contents when compared to forestry materials. These lead to their low-energy production and chemical consumption in delignification methods and fiber treatment. Given the low financial value of the present applications and the high value of potential cellulose products, there is a significant prospect of transforming these biomass resources into value-added products. As the pulp industry monitors the up-to-date trend of pursuing sustainable practices, it will further enhance research on sustainable biomass procurement, advocating circular economy projects to attain environmentally friendly and sustainable development growth [58,59,60]. Therefore, lignocellulosic biomass has been discovered to be a suitable alternative material to wood-derived cellulose for the isolation of cellulose nanocrystals, regarding their abundant accessibility from various resources.

Lignocellulosic biomass is valued as the most widely known biopolymer in the world. The annual production of this biopolymer globally is approximately 1.3 × 10^10^ metric tons [61]. Lignocellulosic biomass comprises agriculture wastes (Figure 3)(corncob, palm residues, straw, empty fruit bunch, sugar cane bagasse, Nile rose, and stover [62,63], forest wastes (branches, unwanted stems, and withered leaves) [64], and industrial wastes (waste paper and demolished wood) [65]. The use of green, renewable and sustainable materials has gradually become more significant for the production of numerous value-added products with low environmental impacts [66,67]. This area of research has attracted the interest of a great number of scholars and industrialists, as such materials are an alternative answer to global warming, environmental pollution, the exhaustion of non-renewable sources, and the energy crisis. 

**Figure 6:**
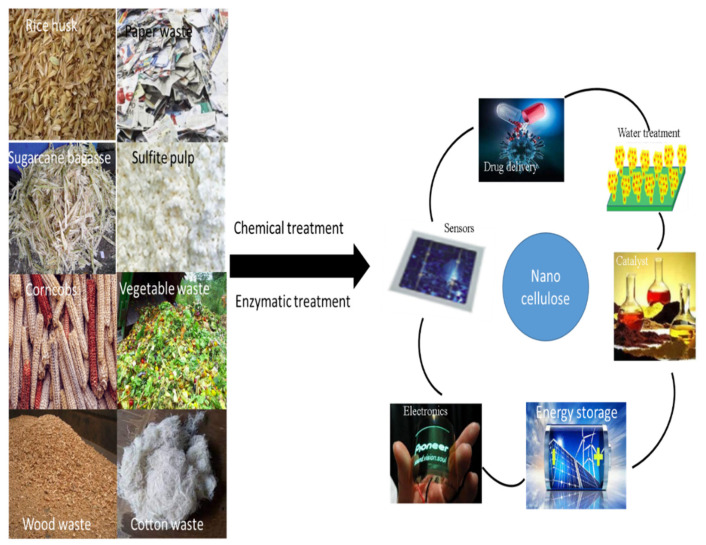
Potential applications of nanocellulose from waste biomass to value-added products (adapted and modified from Thakur et al. [69]).

Within this framework, cellulose, alginate, starch, chitin, gelatin, and chitosan are excellent alternative materials based on their abundant availability from numerous resources [68]. Among others, cellulose is the most abundant renewable compound derived from the biosphere and can be found in algae, plants, bacteria, and some tunicates [21]. Recently, various studies have focused on CNCs’ applications, such as biosensors, energy storage, electronics, antibacterial drug delivery, tissue engineering, membranes, biological imaging, catalysts, and vascular transplantation (Figure 6) [23,24,25]. Nevertheless, further research is required in the area of membrane applications of cellulose nanocrystals derived from waste biomass for wastewater treatment. Different techniques have been employed in the isolation of CNC from lignocellulosic biomass. They are briefly discussed in the next section. 

## 2. Current Techniques for the Isolation of Nanocellulose from Lignocellulosic Biomass Sources

The development of novel nanocellulose-based materials from lignocellulosic biomass has gained the attention of many researchers because of their unique properties, such as easy processing, good thermal stability, nanometric scale, high aspect ratio, nontoxicity, large specific surface area, and remarkable mechanical characteristics of cellulose nanocrystals [70,71]. There are various waste biomass sources available for the isolation of cellulose nanocrystals, and the extraction of cellulose from them involves several processes. They can generally be categorized into agricultural waste and domestic waste (e.g., paper, wood, and plant waste). The approach usually involves chemical techniques such as acid hydrolysis, alkaline bleaching treatment, and chlorination [72]. However, in this review paper, the main focus will be on the extraction of cellulose from agricultural residues and plants (waste lignocellulose biomass, e.g., corncobs, rice husks, sugarcane bagasse, and cashew nut shells, etc.). 

Lignocellulosic biomass is usually pretreated to remove the crystalline parts and leave the crystalline components of the biomass before the CNCs’ isolation. There are several techniques for the pretreatment of lignocellulosic materials to extract cellulose, such as alkaline treatment, steam explosion, and solvent extraction. The conventional extraction techniques can be categorized into chemical methods, physical methods, and enzymatic methods, etc. [21]. The physical methods of preparation involve mechanical grinding, low-temperature treatment, microfluidization technology, and high-intensity ultrasonic treatment, etc. [73]. The sizes of CNCs obtained from the physical method of pretreatment are still large, and the amorphous region is not completely removed. The physical pretreatment methods are also energy-consuming and waste a lot of resources. Therefore, ordinary physical techniques are not often utilized for CNC preparation; rather, they play more of a supporting role [74]. CNC sizes are usually not uniform; therefore, enzymatic hydrolysis reactions have to be repeated many times to break down the larger particles, to ensure uniform CNC sizes, making the process more complex [75]. Chemical techniques are regarded as the major way of preparing high-quality CNCs, and are the main focus in this review paper [21]. There is an improvement in the crystallinity of CNC-based products obtained by acid hydrolysis. This is due to the preferential destruction of the cellulose’s amorphous region during the acid hydrolysis process, leaving rod-shaped crystalline areas [76]. 

### Surface Chemical Modification of CNC

#### Recent Advances in the Chemical Surface Modification of Cellulose Nanocrystals from the Literature

A highly specific surface area (SSA) is crucial for the effective implementation of chemical surface modification, i.e., attaching functional groups to the surface of the CNC [77]. Primarily, cations such as heavy metal ions can be efficiently removed from the water, although by proper modification ammonium groups, negatively charged moieties such as phosphates or nitrates [78,79], and organic pollutants including oils, dyes, pharmaceuticals, and pesticides can also be removed [80,81]. Hydroxyl functional groups are already attracted towards heavy metal ions and dyes [82]; however, the proper modification of OH groups into functional groups with advanced affinity in the direction of charged moieties improves the adsorption or separation capacities by orders of magnitude. For example, CNCs prepared by sulfuric or phosphoric acid hydrolysis characteristically possess anionic groups with a high affinity toward heavy metal ions or dyes [80,83]. The 2,2,6,6-Tetramethylpiperidine-1-oxyl oxidized (TEMPO-oxidized) CNCs carry negatively charged carboxylic groups that attract cations. The utilization of several kinds of modified NC, the attached functional groups, and their affinity for pollutants is currently being studied by many researchers [77,80,81,83,84,85]. Table 1 summarizes the current studies on the surface chemical functionalization of cellulose nanocrystals and their functional groups.

Even though great advancement and progress have been achieved on the surface functionalization and membrane application of CNC-based materials and composites, there are some concerns regarding their preparations. For instance, several methods have been reported for CNCs’ preparation; nevertheless, CNCs’ synthesis with well-regulated size, morphology, and surface characteristics remains a challenge. Furthermore, the ultimate properties and the performance of the CNC-based composite could be influenced by surface modifications; however, the connection between the surface properties and their inherent potential is yet to be known. Lastly, while CNC-based composites have demonstrated great prospects for numerous applications, the dispersion of CNC-based composites in membrane matrixes has not been investigated well. Hence, there is still a large space for the improvement of CNC-based composites for applications in the treatment of wastewater. 

## 3. Membrane Processes for Water and Wastewater Treatments 

In this section, different types of membrane processes—such as reverse osmosis, microfiltration, ultrafiltration, nanofiltration, and membrane distillation—are usually employed for the treatment of water and wastewater, their advantages, and disadvantages are briefly described.

Membrane-based Reverse Osmosis (RO) has received attention over all other existing technologies. Reverse osmosis (RO) is the process of separating dissolved salts from water with the assistance of a semi-permeable membrane that allows the passage of water through the membrane, while the passage of salt is rejected. The saline water is fed to the membranes at high pressure, which passes through the membranes, and salt-free permeate is collected on the low-pressure side. The flux obtained from this membrane is ten times more than the flux obtained from the preceding membranes, with more than 98% rejection efficiency. These make membrane-based reverse osmosis sustainable [97]. The principle of reverse osmosis is dependent on the difference between the water flux and the flux of the salts. The major unavoidable drawback of membrane-based reverse osmosis is fouling. Therefore, it is encouraged that we pre-treat the feed solution before the reverse osmosis operation. Various foulants in the feed solution—such as biofoulant, organic foulant, inorganic foulant and colloidal foulant—can be pre-treated with suitable chemical/physical/biological treatment techniques [98].

Microfiltration (MF) is one of the ancient commercially practiced pressure-driven membrane processes [99]. MF can remove micrometer-sized contaminants—e.g., suspended particles, proteins, bacteria, pathogens, and yeast cells—based on the principle of physical separation [100]. The ability to reject a range of large-scale contaminants makes MF a resourceful membrane technique. The mechanism of separation changes from solution–diffusion (in RO), with an increasing molecular weight of the solute above 500 g.mol^−1^ [101], to molecular filtration in which the separation characteristics are determined by the size of the particle and the pore diameter of the membrane [102]. MF membranes have pore diameters ranging from 0.1 μm to 5 μm. The open membrane structure is employed for the separation of particles with a diameter greater than 0.1 μm. The hydrodynamic resistance being low, such membranes require low hydrostatic pressures for a high contaminant rejection and solvent flux [103]. One of the advantages of MF over other membrane processes is its applicability across numerous areas—including but not limited to pharmaceuticals [104], wastewater treatment [105], food [106], desalination [107], and biotechnology [108] due to their wide range of pore sizes, and it can be operated at low pressure. The enhancement of the MF membrane performance is usually focused on the improvement of the membrane’s permeability, selectivity, resistance to fouling, and cost reduction. These are mainly achieved through the modification of existing membrane materials [100]. Usually, the major challenge of the membrane-based MF is the deposition of various unwanted organic and inorganic species on the surface of the membrane, leading to the blockage of pores, thereby reducing the membrane’s permeability and selectivity. This often results in reduced membrane lifespans. MF membrane process are sometimes used as a pretreatment stage to the RO process. This is often required to increase the resistance of the RO membrane to fouling. Therefore, the urgent development of a novel MF membrane with enhanced properties is required. 

Ultrafiltration (UF) membranes are benign, clean, cost-effective, and effective separation techniques for a wide range of impurities and contaminants in water and wastewater. UF is a membrane filtration method utilized for the mechanical separation of contaminants from a mixture, whereby the solutions are forced through a semi-permeable membrane, aided by the hydrostatic pressure [109]. This separation process separates molecules with a higher molecular weight and suspended solids, which are subject to the molecular weight cut-off (MWCO) indicated by the particular membrane, in conjunction with other factors that can take a significant role, such as the molecule shape, charge, and hydrodynamic conditions [110]. The major mechanism employed in UF is size exclusion; however, the maximum process efficiency might be prevented by the reactions between the particles and the membrane based on the compounds present. The advantages of the UF membrane process over other existing purification processes include but are not limited to its ease of use, the economical advantage of the process due to low energy usage, the high-quality treatment, and the low operating temperature [111]. Membrane fouling is a major hindrance to the successful application of the UF membrane process, especially during the treatment of some complex feeds. One promising way of addressing this drawback could be through the use of functional UF membranes aiming at a definite application [112].

Nanofiltration (NF) membranes have features in between ultrafiltration (UF) and reverse osmosis (RO). NF membranes have a usual pore size of 1 nm, corresponding to the molecular weight cut-off (MWCO) of 300–500 Da. NF membranes in contact with an aqueous solution are also slightly charged due to the dissociation of surface functional groups or the adsorption of the charged solute [113,114]. For example, polymeric NF membranes consist of ionizable groups such as carboxylic groups and sulfonic acid groups, which result in the charged surface in the presence of a feed solution. Like RO membranes, NF membranes are effective for the separation of inorganic salts and small organic molecules. The major exceptional features of NF membranes are the low rejection of monovalent ions, the high rejection of divalent ions, and a higher flux compared to RO membranes. These properties have allowed NF to be employed in various functional applications, such as pharmaceutical, biotechnology, and food engineering applications, and particularly for water and wastewater treatment [114].

Membrane distillation (MD) is a flexible non-isothermal membrane process for separations that is mainly suited for applications in which water is the major component present in the feed to be separated [115,116]. MD refers to the thermally driven transport of vapor molecules through a microporous hydrophobic membrane. Membrane distillation utilizes thermal energy to make provision for a vapor phase of the volatile molecules present in the feed stream (i.e., mostly water), and the condensing of the permeated vapor on the cold side. The driving force in MD is the partial pressure difference between each side of the membrane pores. The temperature difference results in a vapor pressure difference across the membrane. As a result of the hydrophobic nature of the membrane, only vapor can pass across the membrane, and not the liquid solution being distilled [116]. The membrane pore size is usually in the range of 0.1 to 0.5µm [117]. The benefits of the membrane distillation process relative to other membrane separation processes are listed below [118,119]:

i. MD theoretically has a 100% rejection rate for macromolecules, inorganic ions, and non-volatile compounds. Therefore, the same rejection is achieved regardless of the feed concentration.
ii. MD’s operating temperatures are reasonably low compared to other thermal desalination processes that function at elevated temperatures
iii. The operating pressure of the MD process is lower compared to the conventional membrane separation processes such as RO.
iv. The mechanical properties of MD membranes are flexible compared to the existing membrane processes.
v. MD is less prone to fouling and scaling compared to other membrane desalination technologies because it uses a hydrophobic membrane, and the pore sizes are also larger than RO and NF.

**Figure 7:**
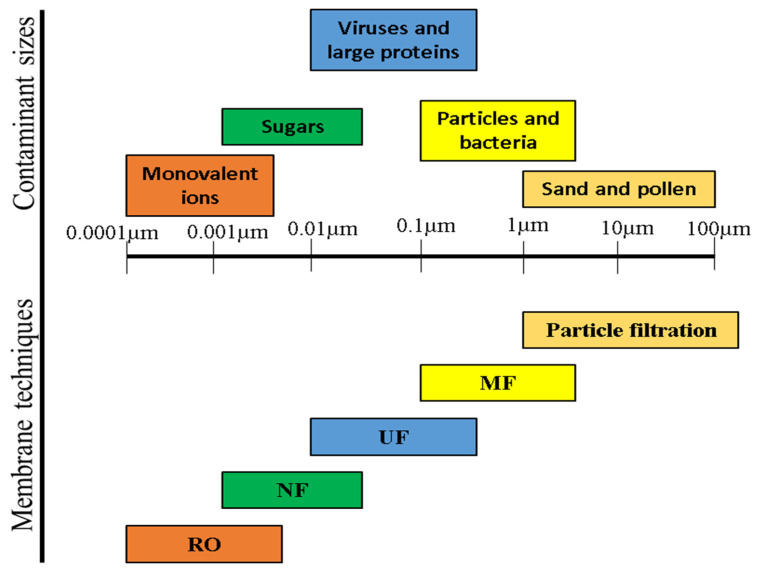
Common membrane-based processes for the removal of contaminants of various sizes from water and wastewater (adapted and modified from Anis et al. [100]).

Figure 7 shows the removal efficiency of prominent membrane-based separation processes including ultrafiltration (UF), nanofiltration (NF), and reverse osmosis (RO) in comparison to MF for various contaminants. 

### 3.1. Potential Applications of Nanocellulose from Waste Biomass in Water Treatment

#### 3.1.1. Cellulose Nanocrystal-Based Polymer Membranes for Wastewater Treatment

A significant benefit of modifying membranes with all-cellulose structures is that they are completely derived from renewable materials, and therefore the disposal of these mainly biodegradable materials is less challenging compared to man-made polymers or composite membranes [29]. Membrane separation is a new method for the removal of pollutants from wastewater without the need for a phase change, except for membrane processes such as membrane distillation and pervaporation, which require a phase change. This method also reduces energy consumption, and separates particles easily from extremely dilute solutions, with higher adeptness [120]. Nanofiltration (NF) membranes and reverse osmosis are commonly used for the removal, recovery, and recycling of valuable or contaminant materials from industrially discharged water. However, NF membranes seem to offer better separation performance as a result of their nanoporous structure, through which most organic matter is unable to pass [121,122]. Recently, the attention of researchers has been drawn towards the application of polymeric membranes for water treatment owing to their benefits such as affordability, high degree of flexibility, direct pore-forming mechanism, and smaller footprint. Nanocomposite membranes comprising dispersed nanomaterials in a polymer matrix show great potential for application in liquid-liquid, gas–gas, and liquid-solid separations. This is attributed to their low cost, biodegradable nature, renewability, improved thermal stability, high specific strength, rigidity, and aspect ratio. 

The incorporation of nanofillers into a polymer matrix is one of the most efficient methods to enhance the properties of a polymer; through this, an improved performance and surface modification of polymer materials are achievable. Examples of such nanofillers are organic, inorganic, metal, and metal oxide nanoparticles [123,124,125]. Cellulose nanocrystals (CNCs) are renewable, degradable, and made from renewable natural cellulose. They are a kind of nano-reinforced nanomaterial that has good mechanical properties, such as a high specific surface area, high crystallinity, high aspect ratio, and high strength, allowing all-encompassing attention in the field of composite materials [126,127]. The two major mechanisms that can be used by nanocomposite membranes are the size exclusion rejection of pollutants based on smaller membrane pore sizes compared to the pollutant, and the adsorption of charged contaminants through electrostatic interaction. One vital pre-step for the development of unmodified nanocomposite membranes which laid down the basics for the theoretical background is the investigation of the cellulose thin films [29]. The development of size exclusion cellulose nano papers was first reported by Metreveli et al. [128] and Mautner et al. [129], showing the contaminant rejection capability of the nanomembrane, down to the 10 nm size of viruses. 

**Figure 8:**
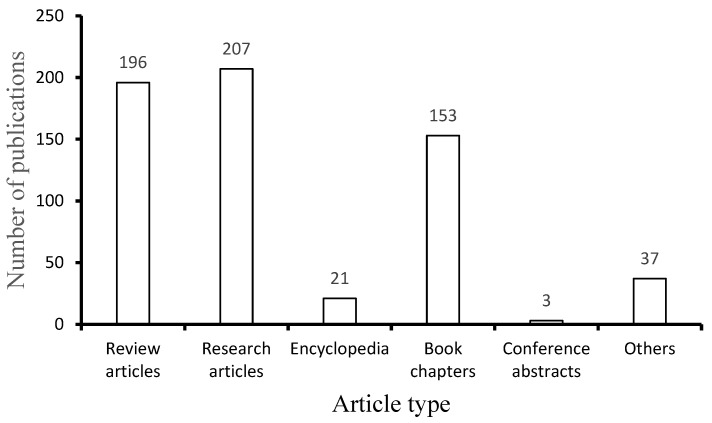
Science direct search engine for nanocellulose crystal-based membranes for wastewater treatment (data extracted from www.sciencedirect.com on 14 July 2021 from Science direct, using the keywords “nanocellulose crystal”, “membranes”, “wastewater”, and “treatment”).

Water pollution from contaminants such as fertilizers, dyes, personal care products, pharmaceuticals, pesticides, metals, metalloids, and so on resulting from domestic, industrial, or agricultural sources poses significant environmental concerns [130,131]. These contaminants are carcinogenic, and usually end up in the water streams, and threaten all kinds of living organisms [130,131]. Hence, numerous techniques have been offered to remove such contaminants, depending on their physical and chemical properties. Cellulose nanoparticles, in particular cellulose nanocrystals (CNC), have been explored in water purification systems due to their high aspect ratio (i.e., the ratio of the length to the width of a particle), mechanical strength, high surface area, and the abundance of chemical groups on their surface, e.g., hydroxyl or carbonyl groups [80]. These functional groups assist with the adsorption of charged contaminants via electrostatic interactions. Cellulose-based materials have shown a remarkably high attraction toward particular contaminants such as metal ions and dyes [132,133,134]. Despite all of the aforementioned benefits, the major challenge in membrane development is the optimization of the balance between porosity, high permeability, and good filtration performance. Therefore, various preparation techniques such as coating, freeze-drying, hybridization, solvent casting, and electrospinning have been utilized for functional membranes [80,135,136]. Figure 8 shows the number of publications (of different types) on the application of different types of cellulose nanocrystal-based membranes in the purification of wastewater.

#### 3.1.2. Recent Advances in Nanocrystal-Based Membranes for Wastewater Treatment

Nanocomposites’ (NCs) application in the treatment of water and wastewater can be achieved through numerous approaches. The utilization of NCs as adsorbent material is the most common approach. Adsorption can be defined as the attachment of contaminants—for example, dyes, heavy metal ions, pesticides, or pharmaceuticals, etc.—onto the surface of a solid material via chemical bonds or electrostatic interaction. The key to a successful implementation of NCs as an adsorbent material is the presence of a high specific surface area (SSA), which provides access to surface functional groups [77]. Therefore, the proper modification of the surface of NCs’ adsorbency could assist in the efficient removal of organic pollutants from water [78,79]. Heavy metal ions and dyes are usually attracted towards hydroxyl groups [82]. However, the further modification of the hydroxyl group into functional groups with higher attraction towards charged ions improves the adsorption capacities by orders of magnitude. For example, CNCs prepared by sulfuric or phosphoric acid hydrolysis inherently possess anionic groups with a high affinity towards dyes or heavy metal ions [80,81,82,83]. 

CNFs modified with oxidized 2,2,6,6-Tetramethylpiperidine-1-oxyl (TEMPO) usually carry negatively charged carboxylic groups that have an affinity toward cations. The further attachment of functional groups that resemble groups frequently used in ion-exchange resins beyond the inherently present functional groups is a possibility [76,137]. In recent times, many researchers have studied the use of different types of modified NC and their attractions for pollutants [77,80,81,83]. 

Globally, domestic water filtration systems are commonly used together with membranes, for effectiveness. This membrane filter is placed at the outlet while utilizing the pressure of the water that flows through the pipe [135]. Surface modification is essential to enhance the dispersion of the nanofiller and the interfacial adhesion between the nanofillers and the polymer matrix [138,139]. De Guzman et al. [140] studied the separation of proteins using a novel CNC-based polymer nanocomposite membrane. The authors initially coated the surface of the CNC with a thin layer of polydopamine (PDA) via self-polymerization to enhance the membrane’s lipophilicity and hydrophilicity. The resultant CNC/PDA was merged with a pore-forming agent and cellulose acetate (CA). The results revealed that the addition of an insignificant amount of CNC/PDA into the membrane (4 wt.%) considerably enhanced the separation performance, tensile strength, and antifouling capability of the CA membrane. The addition of 4 wt.% CNC/PDA also increased the membrane’s permeability, 320 L/(m^2^ h bar), which was 33% higher compared to pristine cellulose acetate. This is an indication that the modification of the CNC with dopamine enhanced the CNC dispersion and the binding force between it and the cellulose acetate. The pore sizes of the fabricated membranes were observed to increase with an increasing amount of CNC/PDA [127,140]. The higher the flux recovery rate (FRR) value, the better the hydraulic cleaning effect of the membrane, the slower the fouling trend of the membrane, and the higher the reusability [141]. FRR is the most direct and simple parameter to measure the antifouling performance of the porous membrane, as membrane fouling significantly affects the permeability of a membrane. An increased FRR value of 77% was obtained for the CNC/PDA/CA membrane, compared to the 67% obtained for the pure CA membrane. The membrane was able to absorb more water molecules, which decreased the adsorption of protein. 

TEMPO-CNFs that carry carboxylic groups were mixed with CTA via the phase inversion technique and tested for protein separation [142]. Reports indicated a 10% reduction in fouling was achieved with FRR of 95%. In addition, the protein rejection and membrane permeance were improved by 30% compared to using a pure CTA without CNF.. The improved antifouling performance could be due to the provision of a high surface charge by the sulfate half-esters in CNCs, which is comparable to the carboxylic groups in TEMPO-CNFs [29]. Likewise, 3-aminopropyl triethoxysilane has also been used to modify the surface of CNCs, and was then incorporated into a PVDF membrane for the removal of dye from wastewater. The membrane permeability improved by 20% compared to an unblended membrane, while the antifouling performance increased by 50% [51]. Similarly, the incorporation of a small amount of CNC (1 wt.%) into the PES polymer membrane enhanced the removal of dye by 10% compared with pristine PES, increased the water flux by three times, and reduced the fouling by 15% [52].

The removal of natural organic matter (NOM) using a nanocomposite membrane was investigated by Bai et al. [143]. In order to reduce the fouling caused by NOM, CNC was blended with PES and fabricated by the phase inversion technique. The physicochemical characterization of the membranes showed that the PES/CNC nanocomposite membrane had higher porosity and zeta potentials compared to the pure PES membrane. An increase in the hydrophilicity of the blended membrane was observed through the static contact angle and the pure water flux of the nanocomposite membranes. However, the CNC loading of 5.0 wt.% significantly reduced the permeability and the porosity of the nanocomposite membranes.

For improved protein removal and polysaccharide adsorption, Lv et al. [144] synthesized graphene oxide-cellulose nanocrystal (GO-CNC) and used it as a novel hydrophilic nanofiller poly(vinylidene fluoride) (PVDF) microporous membrane targeting the enhancement of the antifouling performance of the fabricated GO-CNC/PVDF membrane in a membrane bioreactor (MBR). The physicochemical properties of the GO-CNC composite and the modified PVDF membrane were checked by various characterization techniques, such as scanning electron microscopy with energy dispersive spectroscopy (SEM-EDS), Fourier transform infra-red (FTIR) spectroscopy, and X-ray diffraction (XRD), water contact angle analysis, and zeta potential analysis. The results showed lower protein and polysaccharide adsorption, improved hydrophilicity, and higher permeability compared to CNC/PVDF and GO/PVDF membranes. However, during long-term operation in MBR, the GO-CNC/PVDF membrane displayed a characteristic low EPS accumulation, a higher flux recovery ratio, a lower irreversible fouling, and a longer cleaning cycle due to the improved hydrophilicity, increased porosity, and greater negative zeta potential of the membrane, contributing to the superior antifouling performances displayed by the membrane, with a distinctive higher FRR, longer cleaning cycle, and irreversible fouling property compared to the unmodified PVDF and CNC/PVDF membranes [144]. The improved hydrophilicity displayed by the GO-CNC/PVDF nanocomposite membrane compared to that of the pristine PVDF membrane could be a result of the migration of the hydrophilic CNC and GO-CNC to the surface of the membrane during the phase inversion process. At this point, there is an interaction between the sulfate ester and the oxygen-bearing functional groups of the filler introduced during H_2_SO_4_ hydrolysis and the non-solvent, resulting in improved water adsorption and membrane hydrophilicity [144].

Similarly, Prihatiningtyas et al. [145] synthesized nanocomposite cellulose triacetate/cellulose nanocrystals (CTA/CNCs) for application in a pervaporation desalination membrane. In this study, time-controlled alkaline treatment was intended to increase the water flux of the CTA/CNC nanocomposite membrane without compromising its selectivity. Although there was a drastic improvement in the water flux (107.5 kg·m^−2^·h^−1^) with >99.8% salt rejection compared with the water flux obtained by the unmodified membrane, the membrane water flux was found to be poor compared to the results of other pervaporation desalination membranes reported in the literature on pervaporation desalination membranes. The poor performance of the membrane compared to the perforation membranes in the literature could be due to variations in the particle size, length, and diameter of the cellulose nanocrystals (CNC). Furthermore, it was observed that increasing the initial concentration of NaCl from 90 g/L to 200 g/L decreased the water flux from 107.5 kg·m^−2^·h^−1^ to 58.5 kg·m^−2^·h^−1^. 

The alkaline treatment of the CTA / CNC nanocomposite membrane was studied by Zhang et al. [146] to enhance the absorption of water. Reports showed that water adsorption increased significantly, i.e., by 421%, after 6 min of alkaline treatment compared to the unmodified membrane. However, the further increase of the alkaline treatment time to 20, 30 and 60 min only increased the adsorption water by 36%, 24%, and 13%, respectively. The membrane contact angle was observed to decrease from 65.6° to 26.3° after only 6 min of alkaline treatment. However, there was no significant decrease in the contact angle when the alkaline time increased from 6 to 30 min (26.3 to 24.7°). These results showed that increasing the alkaline treatment time above 6 minutes caused no significant increase in the water uptake or contact angle. Table 2 presents recent studies on the adsorption and membrane performance of CNC-based membranes/adsorbents for the effective removal of pollutants from wastewater. 

In a study by Smith et al. [150], TFNs were manufactured using as-received cellulose nanocrystals (CNC) and 2,2,6,6-Tetramethylpiperidine-1-oxyl (TEMPO) oxidized cellulose nanocrystals (TEMP0-CNC) as the nanoparticle filler. The membrane was fabricated by two methods, namely a vacuum filtration method and a monomer dispersion method, to investigate the influence of nanoparticle dispersion on the membrane flux and salt rejection. Different amounts of CNCs and TOCNs were mixed into the polyamide TFC membrane through in situ interfacial polymerization. The results showed consistency in the TFN formation, with improved water flux and salt rejection for the dispersion method. This is contrary to what was observed for the vacuum filtration method, which resulted in inconsistent TFN formation with poor nanocrystal dispersion in the polymer. Around a 260% increase in water flux and 98.98% salt rejection were obtained for the monomer dispersion method at 0.5 wt.% TOCN loading in comparison to the 97.53 observed for the unmodified polyamide membrane. The increase in water flux by the monomer dispersion method could be due to the formation of nanochannels at the interface between the polyamide matrix and the high aspect ratio of the nanocrystals. These nanochannels serve as rapid transport pathways through the membrane and can be used to alter the selectivity by modifying the interaction between the particle and the polymer [150].

Cutting-edge developments indicate that the utilization of CNCs for wastewater purification tends to be a significant functional filler instead of creating the core structural component. Nanocellulose may still be regarded as a costly material for the main structural and functional component, despite the assumption that NC production on a pilot scale could bring about considerable cost reduction [12]. However, the isolation of CNC from waste biomass could reduce the appreciable cost of NC and address the environmental pollution that contributes to global warming as a result of burning, which is a way of disposing of biomass waste. In addition, the low percentage of CNCs required to enhance the membrane properties would not only lessen the related raw material costs but would also reduce the production times as well, which is a vital factor for the promotion of commercial processes. Attaining uniform and homogeneous dispersion within the polymer matrices is one of the major drawbacks in employing cellulose-based nanocomposites in membranes [12]. Some of the properties of the cellulose, such as the mechanical properties, are lost as a result of the resumption of water sorption mainly at the hydroxyl groups on the CNCs’ surface, arising from the improved membrane hydrophilicity. However, the decrease in the mechanical properties of the membranes may be prevented if natural hydrophilic polymers are used, but this may also affect the membrane permeability. 

Although numerous studies have been carried out recently on the application of membrane technology for the progressive transformation of water and wastewater purification, there is still room for progress in numerous areas. Furthermore, continuous research is required to proffer long-term solutions to membrane application challenges, such as membrane fouling and the high energy demand, through the introduction of tedious but cost-effective pre-treatment techniques, or the fabrication of membranes with antifouling properties.

## 4. Environmental Impact and Life Cycle Assessment of the Application of Cellulose

**Figure 9:**
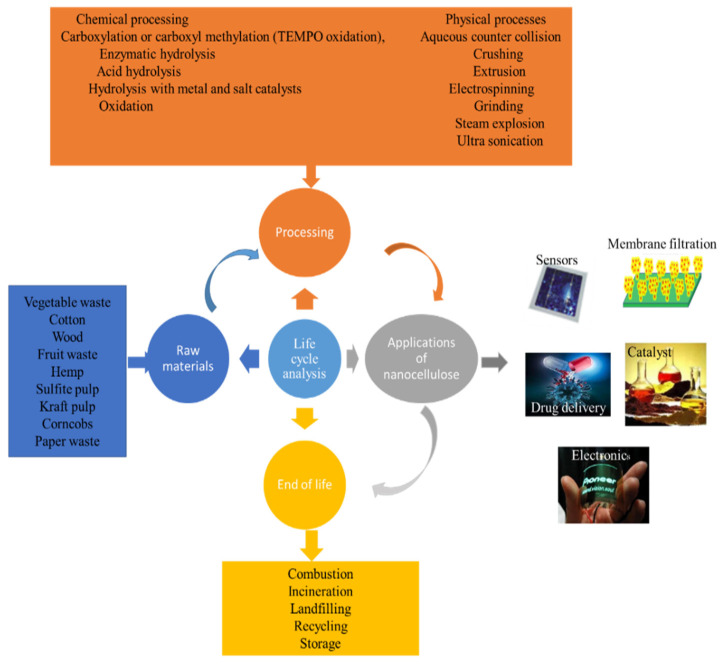
Life cycle analysis of nanocellulose from different waste materials, from the cradle to the grave (modified from Faroughi et al. [151]).

Life Cycle Assessment (LCA) is a suitable instrument for the measurement of the impacts of the environment associated with raw material extraction, manufacturing, the utilization of end products, and disposal [151]. Therefore, LCA is constituted by all the phases, from the fabrication of the products using raw materials (cradle) to the end-of-life (EOL) disposal approach of end products (grave). As a result of the growing application of cellulose products recently, environmental impacts arising from the cellulose extraction, cellulose product fabrication processes, cellulose product utilization, and their end-of-life disposal are evaluated. Efforts should be made to reduce the energy demand and develop a low carbon economy for cellulose products due to challenges like inaccessibility of data, thoughtlessness toward end-of-life treatments, restrictions related to lab-scale processes, and limited LCA research related to cellulose products [151]. In view of this, life cycle analysis (LCA) is a powerful tool for the evaluation of the overall environmental impact ascribed to all of the steps, including the extraction, manufacturing, and use of cellulose, in other words, from cradle to grave. Therefore, appropriate measures must be put in place to mitigate the environmental impact and develop low-carbon cellulosic materials [151,152,153]. Figure 9 shows a schematic of all of the stages of the life cycle of nanocellulose products.

The evaluation of the life cycle of cellulose products has become imperative due to the increasing attention focused on the development of sustainable and renewable nanocellulose. Life cycle analysis can be utilized as a vital instrument for the assessment of environmental impacts. Nevertheless, there is still a lack of accurate evaluation due to insufficient laboratory data that encompass all of the impacts of cellulose products on different environmental aspects, such as human health, air emissions, and the discharge of the waste stream. Furthermore, most laboratory-scale processes do not account for the end-of-life stage of cellulose materials [154,155]. In view of this, production process scale-up is one of the greatest drawbacks, due to wide differences between laboratory and industrial processes [156].

## 5. Challenges and Future Perspectives in the Development of CNCs for Membrane Application in Wastewater Treatment

This paper offers a viewpoint that cellulose nanocrystals can be a significant, environmentally benign, and economically functional novel nanomaterial that is mainly apt for membrane applications in the treatment of water and wastewater. In addition to the provision of efficient and inexpensive platforms to advance commercial-scale water treatment techniques for advanced countries, more notably, these technologies may also offer viable solutions to handle various challenges of off-grid potable water in underdeveloped countries, as well as the treatment of wastewater from various industries.

Although the existing and emerging applications of cellulose nanocrystals for water and wastewater treatment discussed in this review offer evidence that water purification is feasible at a laboratory scale during membrane filtration, further industrial-scale application is required to assert its efficiency and the applicability of the remarkable number of research reports that have been published for wastewater treatment using nanocellulose crystal-based membrane. Some drawbacks still need to be overcome in order to completely comprehend the prospect of most of the evolving applications emphasized in this work at an industrial scale. Insofar as it could be established, most of the wastewater treatment technologies that were reviewed in this paper were used in laboratory-scale situations, and are yet to be used on a large scale. To accelerate the industrialization of the emerging wastewater treatment using the CNC-based membranes reviewed in this paper, stakeholders involved in membrane technology and water management—such as the policymakers, municipalities, researchers, and industries—should have a detailed guideline that consists of the research and design (R&D), industrialization, and application stage. In this way, the challenges of membrane fouling during water purification can be addressed at the commercial scale.

Nanocellulose-based membranes have been evaluated to be an efficient media for pressure-driven filtration processes, such as MF, [157], UF [158], and NF [159]. It is envisioned that nanocellulose membranes can similarly be utilized in the concentration-driven forward osmosis process, where the high flux advantage in nanocellulose membranes may further reduce the consumption of energy, and in the thermally driven membrane distillation procedure, where the reduced cost of nanocellulose membranes may promote the broadcast of technology for the purification of water in rural communities using solar power [160]. With the cost-effective production technique for the isolation of cellulose nanocrystals, integrated water purification techniques that combine coagulation, adsorption, and membrane filtration processes using nano cellulosic materials could be explored. This integrated technique would assist deprived societies using their locally available materials and sustainable technologies to provide accessible and clean drinking water. 

Furthermore, there is a trade-off between improved hydrophilicity and improved mechanical strength relating to the mixing of CNCs into polymer membranes [161]. The rapid degradation of the membrane is a major concern, most especially for membranes that interact easily with bacteria, even though the biodegradability of nanocellulose is a great benefit in numerous applications. However, the degradation of CNs may be avoided if they are stably incorporated into the polymer of the membrane. Polymers would perhaps prevent cellulose from easy degradation; however, further research is needed to establish this fact [12]. Although numerous studies have been carried out recently on the application of membrane technology for the progressive transformation of water and wastewater purification, there is nevertheless still room for progress in numerous areas. Furthermore, continuous research is required to proffer long-term solutions to challenges, such as membrane fouling and high energy demand, associated with membrane applications, through the introduction of tedious but cost-effective pre-treatment techniques, or through the fabrication of membranes with antifouling properties.

## 6. Conclusions

The membrane applications of cellulose nanocrystals derived from lignocellulosic biomass for water and wastewater treatment were extensively reviewed in this work. This paper established that cellulose nanocrystals can be a significant, benign, and economically functional novel nanomaterial that is mainly suitable for membrane applications in the treatment of water and wastewater. In terms of effectiveness, CNC membranes are approaching the development of commercial membranes, particularly when small amounts of CNCs or very thin layers are used. Furthermore, the utilization of waste material for the preparation of cellulose nanocrystals could promote the development of a low-cost integrated water treatment process that incorporates adsorption, coagulation, and membrane filtration. This could be an emerging technique for the effective treatment of water and wastewater. Therefore, the following conclusions can be drawn from this review:

- Waste biomass application as raw materials in the synthesis of cellulose nanocrystals could assist in the attainment of zero-waste generation goals, which are otherwise regarded as a circular bio-economy, alleviating waste management challenges and environmental problems.
- The application of nanotechnology knowledge in the fabrication of nano-enabled membranes is expected to address the drawbacks of existing materials and processes, as well as optimizing the processes’ performance in wastewater treatment applications.
- Even though CNC-based composites have displayed great potential for numerous applications, the dispersion of CNC-based composites in membrane matrixes has not been well studied. Hence, there is still a large space for the development of CNC-based composites for wastewater treatment applications.
- Furthermore, continuous research is required to proffer long-term solutions to challenges, such as membrane fouling and high energy demand, associated with membrane applications through the introduction of tedious but cost-effective pre-treatment techniques, or through the fabrication of membranes with antifouling properties.
- The lack of accurate evaluation due to insufficient laboratory data that encompass all of the effects of cellulose products on different environmental aspects such as human health, air emissions, and the discharge of the waste stream has adversely affected the evaluation of the life cycle of cellulose products.

The following recommendations are provided for future research interest in this area:

- Future investigation should be tailored towards the economic and cost analysis of nanocellulose crystals from agro-waste, comparing them with CNC obtained from wood pulp, to establish their cost-effectiveness if incorporated into membranes for water and wastewater treatment on a commercial scale.
- Lastly, future research should focus on the extension of process integration-based techniques for energy efficiency that combine adsorption, coagulation, and membrane filtration in one system.
- Because the application of water purification process integration concepts has not been satisfactorily explored, pollutants such as phenol, benzene, toluene, ethylbenzene, and xylene (BTEX) compounds can be efficiently removed from water and wastewater treatment.
- More studies and comprehensive reviews are also required in membrane applications of cellulose nanocrystals for the treatment of water and wastewater to aid the trend of the lab-scale development of CNC-based membranes at the industrial scale with a good market value.
- Furthermore, the lab-scale processes must account for the end-of-life stage of the cellulose materials to provide a proper assessment for the life cycle of cellulose products.

## Figures and Tables

**Table 1:** Recent studies on the surface chemical functionalization of cellulose nanocrystals and their functional groups.

Cellulose Type	Functionalization Chemical	Pollutant	Attached Functional Group	Properties	Ref.
CNC	Polydopamine Melanine-formaldehyde	Methylene blue (MB), Methyl orange (MO) Crystal violet (CV)	Cationic amine groups	-	Mohammed et al. [9]
CNC	Maleic acid		Carboxylated	Stronger and tougher films	Wang et al. [86]
CNC	Maleic anhydride	Multiple cationic dyes	Carboxylate	Maximum cationic dye uptake	Qiao et al. [87]
CNC	Ethylene diamine	Diclofenac sodium	Amino group	Adsorption capacity of 444.44 mg/g	Hu et al. [88]
CNC	Potassium Carbonate	Vinyl acetate	Acylate group	Influenced Chemical Activity Enhanced dispersion	Brand et al. [89]
CNC	Plasma-induced argon/methane (Ar/CH4), argon/ammonia (Ar/NH3) and argon/silane (Ar/ SiH4).	-	SiO_2_ bonds O-C=O/N-C=O C-C/C-H	Promotes hydrophobicity and hydrophilicity	Matouka et al. [90]
CNC	Palmitoyl chloride and ε-caprolactone	poly(β-hydroxybutyrate-co-valerate	Surface grafting	Improved tensile strength	Chen et al. [91]
CNC/ poly(methyl methacrylate)	Malic acid		Carboxyl groups	Stronger and tougher films	Wang et al. [86]
CNC	Evaporation-induced self-assembly (EISA). cobalt ferrite (CoFe_2_O_4_)	-		Good dispersibility	Lizundia et al. [92]
CNC	Chitosan cross-link with glutaraldehyde	Dyes Victoria Blue Methyl violet Rhodamine		Victoria blue 98%, methyl violet 84%, Rhodamine 70%	Karima et al. [93]
CNC	Hydrolysis: Sulfuric acid (5–10%) Esterification: Acetic acid (70–90%)	-	Carboxyl	Excellent dispersion High thermal stability	Wang et al. [94]
CNC	Acid hydrolysis Anion exchange: sodium dodecylbenzene sulfonate.	-	Non-covalent interaction	Improve dispersion Integrated chemical structure	Huang et al. [95]
CNC	H_2_SO_4_/Oxalic acid hydrolysis.	-	Carboxyl group	Good thermal stability Improved dispersion	Xie et al. [96]

**Table 2:** Recent studies on the adsorption and membrane performance of CNC-based membranes/adsorbents for the effective removal of pollutants from wastewater.

Composite	Contaminant	Removal Efficiency (%)	Ref.
PES/1%MCNC	Copper ions	90	Rafieian et al. [52]
Double layered GO/CNF membranes	Victoria blue 2B Methyl violet 2B	98.8 97.6 92.3	Liu et al. [136]
CTA/CNC	Saline solution	99.8	Zhang et al. [146]
CNC/PES	BSA	93	Zhang et al. [146]
Amine functionalized CNCs	Acid red GR	555.6 mg/g	Jin et al. [147]
PES/1%MCNC	Coloured dissolved compounds from the licorice processing industry	94.2	Jonoobi et al. [148]
CNC based nanofiltration membrane	NaSO_4_ Mg_2_SO_4_	98 96	Huang et al. [149]

BSA, bovine serum albumin; CTA, cellulose triacetate; PDA, polydopamine.

## Data Availability

Data sharing does not apply to this article.
